# Baseline Assessment of a Healthy Corner Store Initiative: Associations between Food Store Environments, Shopping Patterns, Customer Purchases, and Dietary Intake in Eastern North Carolina

**DOI:** 10.3390/ijerph14101189

**Published:** 2017-10-07

**Authors:** Stephanie B. Jilcott Pitts, Qiang Wu, Kimberly P. Truesdale, Melissa N. Laska, Taras Grinchak, Jared T. McGuirt, Lindsey Haynes-Maslow, Ronny A. Bell, Alice S. Ammerman

**Affiliations:** 1 Department of Public Health, Brody School of Medicine, East Carolina University, Greenville, NC 27834, USA; grinchakt13@students.ecu.edu (T.G.); bellr16@ecu.edu (R.A.B.); 2 Department of Biostatistics, College of Allied Health, East Carolina University, Greenville, NC 27834, USA; wuq@ecu.edu; 3 Department of Nutrition, University of North Carolina at Chapel Hill, Chapel Hill, NC 27514, USA; Kim_Truesdale@unc.edu (K.P.T.); alice_ammerman@unc.edu (A.S.A.); 4 Division of Epidemiology and Community Health, University of Minnesota, Minneapolis, MN 55455, USA; nels5024@umn.edu; 5 Department of Nutrition, University of North Carolina at Greensboro, Greensboro, NC 27413, USA; jtmcguir@uncg.edu; 6 Department of Agricultural and Human Sciences, North Carolina State University, Raleigh, NC 27695, USA; lhmaslow@ncsu.edu

**Keywords:** food environment, diet, food availability, food store, convenience store

## Abstract

In 2016, the North Carolina (NC) Legislature allocated $250,000 to the NC Department of Agriculture, to identify and equip small food retailers to stock healthier foods and beverages in eastern NC food deserts (the NC Healthy Food Small Retailer Program, HFSRP). The purpose of this study was to examine associations between food store environments, shopping patterns, customer purchases, and dietary consumption among corner store customers. We surveyed 479 customers in 16 corner stores regarding demographics, food purchased, shopping patterns, and self-reported fruit, vegetable, and soda consumption. We objectively assessed fruit and vegetable consumption using a non-invasive reflection spectroscopy device to measure skin carotenoids. We examined associations between variables of interest, using Pearson’s correlation coefficients and adjusted linear regression analyses. A majority (66%) of participants were African American, with a mean age of 43 years, and a mean body mass index (BMI) of 30.0 kg/m^2^. There were no significant associations between the healthfulness of food store offerings, customer purchases, or dietary consumption. Participants who said they had purchased fruits and vegetables at the store previously reported higher produce intake (5.70 (4.29) vs. 4.60 (3.28) servings per day, *p* = 0.021) versus those who had not previously purchased fresh produce. The NC Legislature has allocated another $250,000 to the HFSRP for the 2018 fiscal year. Thus, evaluation results will be important to inform future healthy corner store policies and initiatives.

## 1. Introduction

A diet rich in fruits and vegetables is associated with lower rates of obesity [1,2,3], some cancers [4], cardiovascular disease [5,6], type 2 diabetes [7], and all-cause mortality [8,9]. However, in general, Americans do not consume recommended amounts of fruits and vegetables [10], particularly those in rural [11] and disadvantaged [12] populations. In addition, residents of rural and underserved areas are more likely to be affected by obesity, compared to their urban counterparts [13,14]. Thus, developing and testing strategies to increase fruit and vegetable consumption and reduce obesity is important to prevent and reduce diet-related chronic disease disparities.

The social ecological model posits that healthy behaviors can be facilitated by considering individual, interpersonal, organizational, community, and policy levels of influence [15]. Glanz et al. [16] encouraged examination of both the community food environment (food retail venues in one’s neighborhood) and consumer food environment (foods and beverages offered within each food venue) in epidemiologic and intervention research [16]. Thus, healthy small food store or corner store initiatives have proliferated as potential solutions to increase healthy food access and consumption, especially among disadvantaged populations [17,18]. Promoting healthy foods and beverages in corner stores can positively impact population-level dietary behaviors, since corner stores often stock and promote unhealthy foods and beverages, and customers frequently purchase these unhealthy foods and beverages [19,20]. Healthy small store initiatives have been successful at promoting healthier dietary practices and intentions in numerous studies, though others have not shown changes in dietary practices and shopping behaviors [17,18,21]. These mixed results could be due to inaccurate and inconsistent measurement of store purchases and dietary consumption. Studies of associations between food store environments, customer purchases, and dietary intake have been conducted in supermarket settings [22,23], with findings indicating that the price of food and beverage options in supermarkets is associated with food consumption and body mass index (BMI) [22,23].

A few researchers have investigated the association between store purchases and healthfulness of store offerings: Caspi et al. [24] found that Healthy Eating Index scores of aggregate store purchases were associated with fruit and vegetable shelf space, and offering at least 14 varieties of fruits and vegetables was associated with produce purchases. Martin et al. [25] found that each additional type of fruit or vegetable available at a small store was associated with 12–15% higher odds of customers purchasing a fruit or vegetable. To determine whether providing and promoting healthier food and beverage options in small store food environments is worth the public investment that states and municipalities are making, it is important to understand how the food environment in small stores is related to customer purchases, and in turn, how these elements are related to dietary intake among customers. However, to our knowledge, no studies to date have linked the healthfulness of the foods and beverages present in small stores, healthfulness of customer purchases, and objectively-assessed fruit and vegetable consumption. More work is needed to evaluate healthy corner store initiatives using objective measures of purchases and dietary behaviors, due to extensive measurement error in self-reported dietary assessment [26].

Shopping patterns, and shopping frequency in particular, are critical areas of study with regard to the food environment, as the dose of the exposure of interest (the store’s consumer food environment) is determined by how often a customer shops in that store. Determination of dose is vital to learning more about how elements of the food environment might be associated with purchases and consumption. For example, in an evaluation of a local farmers’ market initiative, Jilcott Pitts et al. [27] found that those who reported shopping more frequently at farmers’ markets also reported higher fruit and vegetable consumption, potentially suggesting a dose–response relationship. It is important to examine whether similar relationships might be found among customers in corner stores, particularly in the rural food environment, as rural residents might be more likely to rely on small corner stores for regular food and beverage needs [28].

Therefore, the purpose of this study was to examine associations between the healthfulness of food store environments (food and beverage availability, price and quality), shopping patterns, customer purchases, self-reported diet, and objectively measured fruit and vegetable consumption among customers in corner stores in eastern North Carolina. This study served as baseline data collection for an evaluation of the North Carolina Healthy Food Small Retailer Program (NC HFSRP) on dietary behaviors among residents of eastern North Carolina. For this study, we asked the following research questions: (1) What are the associations between the healthfulness of foods and beverages offered in stores, customer purchases, and customers’ dietary intake? (2) What are the associations between shopping patterns and customers’ dietary intake? We assessed shopping patterns by determining frequency of shopping at the corner store and previous purchase of fruits or vegetables at the store. We used two measures of dietary intake: self-reported fruit, vegetable, soda and sweetened fruit drink consumption; and a novel, non-invasive objective method of fruit and vegetable consumption, using a reflection spectroscopy device (RS Device, the “Veggie Meter” (Longevity Link Corp.™ Salt Lake City, UT, USA)) to measure skin carotenoid status.

## 2. Materials and Methods

### 2.1. Setting and Context

In July 2016, and again in July 2017, the NC Legislature allocated $250,000 each year ($500,000 total) for the creation of a HFSRP in eastern NC. HFSRP funds are to be used to reimburse small food retailers (<3000 heated square feet) for the purchase and installation of refrigeration equipment and other equipment necessary for stocking nutrient-dense foods, including fresh vegetables and fruits, whole grains, nuts, seeds, beans and legumes, low-fat dairy products, lean meats, and seafood. Eligible retailers must meet square footage requirements, be willing to accept (or already accept) Supplemental Nutrition Assistance Program (SNAP) benefits and Special Supplemental Nutrition Program for Women, Infants, and Children (WIC) benefits, and be located in areas that qualify as food desert zones, according to the Economic Research Service of the United States Department of Agriculture [29]. As part of a separate and independent evaluation effort, we collected baseline data on the foods and beverages available at each store, customer purchases, and conducted a customer intercept survey among 479 customers in 16 eastern NC corner stores. The East Carolina University Institutional Review Board reviewed and approved this study (UMCIRB 16-002420), and participants received a $10 gift card for participation.

### 2.2. HFSRP and Control Stores

The NC Legislature appointed the NC Department of Agriculture and Consumer Services (NCDACS) to serve as the HFSRP implementing agency, and stores were selected using a competitive application process. The NCDACS received 17 applications. Based upon communication from the NCDACS, of the 17 corner stores that applied, three were not interested at follow up (no reason given), two were not confident they could be successful at selling healthier options, four misunderstood the application and the HFSRP, and two were not located in a food desert. Thus, a total of six stores enrolled; in the HFSRP, in Bertie, Pasquotank, Onslow (2 stores), and Bladen (2 stores) Counties. One of the six HFSRP stores did not wish to participate in the evaluation, leaving five HFSRP stores in this analysis.

Control stores were selected from counties in eastern NC that were similar to HFSRP store counties, based on geographic proximity (a proxy for cultural similarity). Potential control stores in those counties were identified using the Reference USA business database, with convenience stores and small grocery stores (including information on store size) extracted, based on matching North American Industrial Classification System (NAICS) codes and census data. The selected matching variables included: (1) NAICS designated store type, (2) store size (1499 and below, 1500 to 2499, and 2500–4999), (3) census tract level food desert type (access defined by income, vehicle access, and distance), (4) percent households receiving Supplemental Nutrition Assistance Program (SNAP), and (5) percent African American (tract level). We matched control stores to HFSRP stores, starting with variables deemed most important, in the following order: (1) store type, (2) store size, and (3) census tract level food desert type. We then ordered the remaining matching control stores for each HFSRP store by the smallest differences in values for percent of households receiving SNAP, and then took the remaining closest matches (5–10) and ordered them by closest match with HFSRP store census tract percent African American. Finally, we contacted stores on the final list, starting with the closest match, to see if they were willing to participate as controls. A total of 11 stores were confirmed as suitable controls.

### 2.3. In-Store Assessments

To ascertain the types of foods and beverages in each store at baseline, in-store assessments were conducted using an adapted version of the validated Nutrition Environment Measures Survey for Stores (NEMS-S), used in previous corner store studies [19,30,31]. The adapted NEMS-S measures availability, price, and quality for 18 foods/food types. When conducting the audits in the first two corner stores, the two independent observers (graduate research assistants) achieved 93–95% agreement. We replicated the method of Caspi et al. [19] to create a Healthy Food Supply (HFS) score, to summarize availability, price, quality, and variety of food and beverage items in each store, as described previously [31]. The HFS score has a possible range of 0–31, with higher scores indicating healthier items. The goal was to visit each store twice and calculate a separate HFS score for each visit. For the five HFSRP stores, each store was visited twice, and different HFS scores were calculated for each visit. Seven control stores had two HFS scores, three were missing one HFS score, and one was missing both HFS scores (but customer intercept surveys were conducted at that store) (See Table 1).

### 2.4. Customer Intercept Surveys and Bag Checks

To assess customer food and beverage purchases, customers were approached after exiting the store with observable store purchases. Interested customers were screened for eligibility (over 18 years of age, purchased a food or beverage item, and an English speaker). If both interested and eligible, respondents completed a customer intercept survey, which included a bag check and customer questionnaire.

### 2.5. Customer Purchases

Most customers who completed a questionnaire also completed a bag check. We used the methods used in prior studies to conduct bag checks [19,20], wherein each interviewer examined items purchased during the store visit. Interviewers recorded detailed information on each food and beverage item purchased, including the product name, brand, size, quantity, and price paid. The National Cancer Institute Automated Self-Administered 24 h recall (ASA24) website and nutrient database were used to determine kilocalories, added sugars, fiber, and general nutrient profile of purchases made. The purchases from each store’s customers were combined and entered as one food record on the ASA24. We then calculated the Healthy Eating Index-2010 (HEI-2010) scores for purchases using the SAS macros provided by NCI (https://epi.grants.cancer.gov/asa24/resources/hei.html). The HEI-2010 is a valid indicator of whether a diet or food source is consistent with federal dietary guidelines, [32,33], and has been used previously to assess the healthfulness of food store purchases [19]. We computed store-level HEI from the combined customer purchases as gathered from the bag checks. Thus, we computed one HEI score per store per day. A higher score indicated that customers from that store had healthier food purchases.

### 2.6. Self-Reported Dietary Intake

After completing the bag check, customers were asked to complete a questionnaire to assess dietary intake, shopping patterns, and demographic characteristics. The questionnaire included self-reported fruit and vegetable consumption using the National Cancer Institute (NCI) Fruit and Vegetable Screener [34]. The NCI screener includes frequency and amount questions for the following items: 100% juice (orange, apple, grape, or grapefruit); fruit (fresh, canned, frozen, no juice); lettuce salad; French fries/fried potatoes; other white potatoes (baked, boiled, and mashed potatoes, potato salad, and white potatoes that were not fried.); cooked dried beans; other vegetables; and tomato sauce (tomato sauce on pasta or macaroni, rice, pizza and other dishes). It also includes frequency for vegetable mixtures (foods such as sandwiches, casseroles, stews, stir-fry, omelets, and tacos). Servings per day were calculated based upon the NCI’s standard scoring algorithms found at https://epi.grants.cancer.gov/diet/screeners/fruitveg/scoring/allday.html#how. Participants were also queried about frequency of “regular soda” consumption (“do not include diet soda or diet pop”) and “sweetened fruit drink consumption” (including Kool-aid, cranberry, and lemonade. Include fruit drinks you made at home and added sugar to.” Response options included never; 1–3 times/month; 1–2 times/week; 3–4 times/week; 5–6 times/week; 1 time/day; 2 times per day; 3 times per day; 4 times per day; and more than 5 times per day. These items were adapted from the Behavioral Risk Factor Surveillance System survey [35]. We focused on fruits, vegetables, and sugary beverages because fruits and vegetable indicate a healthier dietary pattern, in most cases, and sugary beverage consumption indicates a less healthy dietary pattern. In addition, these foods and beverages are common targets of interventions. Finally, we were limited on time, and could not burden our participants with too many dietary measures, and so we tried to select the most salient measures for our intervention.

### 2.7. Shopping Patterns

Customers were asked if they had ever bought fruits and vegetables at the corner store (yes or no). Participants reported the “dose” of shopping frequency, with response options ranging from more than one time per day to less than one time per month. Due to the distribution of data, for analyses, responses were dichotomized into those who shopped at least once per day, and those who shopped five–six times per week or less.

### 2.8. Demographics

The questionnaire also included questions to ascertain age (in years), sex (male, female, other), race/ethnicity (American Indian or Alaskan Native, Asian, Black or African American, Native Hawaiian or Pacific Islander, Hispanic or Latino, White, Other or Refused), and self-reported weight and height. Body mass index (BMI, kg/m^2^) was calculated from self-reported height and weight.

### 2.9. Objective Measure of Fruit and Vegetable Intake

Skin carotenoid status assessed by resonance Raman spectroscopy (RRS), has emerged as a relatively novel biomarker of fruit and vegetable intake [36]. A wide distribution of skin carotenoid levels has been demonstrated [37,38], with high reproducibility over six months [38,39] and validity compared to plasma, skin biopsy, and reported fruit and vegetable intake [39]. Reflection spectroscopy (RS) is highly correlated with RRS (*r* = 0.85), [40], and can be measured using a small, portable device, easily set up in field settings (the RS Device, or “Veggie Meter”). Therefore, in addition to self-reported dietary assessment, we also used the RS Device to objectively assess skin carotenoids as an objective measure of dietary intake of fruits and vegetables [41].

Skin carotenoids have been used as a biomarker of fruit and vegetable consumption in prior studies [36,37,38,39,42,43]. To measure skin carotenoids in this study, individuals were asked to place their index finger on a lens on the top of the instrument, as the index finger is an optimal place for assessment of skin carotenoids [44]. A lever was then lowered over the finger, which applied a gentle pressure. The device was linked to a tablet computer, and showed the participant where his or her skin carotenoid reading falls on a histogram of all other readings prior to that date [41]. RS readings range from 0 to 800, with a higher score indicating greater skin carotenoids.

### 2.10. Data Analysis

Audits, customer intercept surveys and carotenoid assessment using the RS Device were conducted at baseline in each store, with the goal of 30 customer intercept surveys per store, which was the amount estimated for adequate power to detect change in fruit and vegetable consumption between baseline and follow-up for the NC HFSRP evaluation. For this study, we examined the store-level HFS score and store-level HEI score for all bag checks conducted in each store. In order to examine associations between store-level HFS score (healthfulness of foods and beverages offered in the stores), store-level HEI (customer purchases), and self-reported and objectively measured fruit and vegetable consumption (via the RS Device), soda and sweetened fruit drink consumption (customers’ dietary intake), we used Pearson’s correlation coefficients and multiple linear regression analyses, controlling for age, sex, and race. We examined associations between shopping patterns and consumption by comparing consumption among those who said they had purchased fruits and vegetables at the store before, versus those who did not, using *t*-tests and adjusted linear regression analyses. We also examined food consumption patterns and BMI based upon frequency of shopping at the store using adjusted linear regression analyses. SAS version 9.4 (SAS Institute, Cary, NC, USA) was used for all analyses.

## 3. Results

### 3.1. Store and Customer Characteristics

Table 1 includes the HFS and HEI scores for five HFSRP stores and eleven control stores at baseline. The HFS scores ranged from 0.5 to 11, and HEI scores ranged from 18.8 to 60.6.

Table 2 includes participant demographics, and shows that a majority (66%) were African American, with a mean age of 43 years, a mean RS reading of 234 intensities, a mean daily self-reported intake of fruits and vegetables of 2.2 (fruit) and 2.7 (vegetables), and a mean BMI of 30.0 kg/m^2^. Control store customers were not significantly different from HFSRP customers on age, sex, BMI, fruit and vegetable consumption, or RS scores. Control store customers were more likely to be African American than HFSRP customers (*p* < 0.001), and control store customers shopped more frequently at the store than did HFSRP customers (*p* < 0.001). The Coefficient of Variation for the RS Device was lower among HFSRP versus control customers (5.5 versus 7.3%, *p* = 0.01). Control store customers also reported higher soda and sweetened fruit drink consumption compared to HFSRP customers. The store-level HFS score for control stores was 4.26 (standard deviation = 2.47) and for HFSRP stores was 4.85 (2.47), *p* = 0.56). The HEI for control stores was 35.5 (standard deviation = 7.4) and for HFSRP stores was 43.6 (11.8), *p* = 0.070.

### 3.2. Associations between Store-Level HFS Score, Store-Level HEI and Dietary Consumption

The correlation between HEI score and carotenoids as assessed using the RS Device was in the expected direction, though not statistically significant [*r* = 0.087, (95% CI, −0.009, 0.182) *p* = 0.076]. There were no statistically significant correlations between HFS scores, HEI scores, and dietary consumption (Table 3).

In models (adjusted for age, sex, and race), there were no associations between HFS score as the independent variable and self-reported fruit and vegetable consumption, carotenoids (RS Device Intensities), soda, or sweetened fruit drink consumption. There were also no associations between HEI and self-reported fruit and vegetable consumption, carotenoids, and soda consumption. There was an inverse association between HEI and sweetened fruit drink consumption [parameter estimate = −0.012 (0.007), *p* = 0.074, 95% CI = (−0.26, 0.001)] (Table 4).

### 3.3. Shopping Patterns and Dietary Consumption

Those who said they had purchased fruits and vegetables at the store previously reported higher fruit and vegetable intake (5.70 (4.29) vs. 4.60 (3.28), *p* = 0.021, 95% CI for difference is (0.169, 2.033) compared to those who had not previously purchased fruits and vegetables at the store, whereas those who said they had previously purchased fruits and vegetables from the store had lower carotenoid values (225.3 (78.0) vs. 237.1 (88.6), *p* = 0.194, (−29.6, 6.06)). The association between self-reported fruit and vegetable consumption and prior store fruit and vegetable purchases remained significant in multivariable linear regression (−1.295 (−2.120, −0.471), SE = 0.420, *p* = 0.002), and there was no association between carotenoids and prior store fruit and vegetable purchases in multivariable linear regression (−5.39 (−13.7, 24.5), SE = 9.72, *p* = 0.579).

Those who shopped at the store one time per day or more reported 5.08 (3.68) fruits and vegetables consumed daily, versus those who shopped five–six times/week or less, who reported 4.63 (3.39) fruits and vegetables daily (95% CI for the difference = −0.236, 1.142, *p* = 0.197). There was also no difference between carotenoids and frequency of shopping (95% CI for the difference = −13.3, 20.7, *p* = 0.673). Those who shopped less frequently had a slightly higher mean BMI than those who shopped more frequently (30.8 (8.54) vs. 29.5 (7.39), 95% CI for difference = −0.187, 2.896, *p* = 0.085). Customers who reported greater shopping frequency had higher soda consumption than those reporting less frequent shopping. (1.49 (1.72) vs. 1.18 (1.55), 95% CI for difference = −0.008, 0.619, *p* = 0.056).

## 4. Discussion

In their study of the influence of a new supermarket on the surrounding food environment, Ghosh-Dastidar et al. [45] concluded that “*it will be important for healthy food initiatives to consider the total retail environment …. This might mean including supports for other stores to maintain or increase their healthy offerings in the face of new supermarkets or doing so instead of introducing new supermarkets*”. The NC HFSRP is one such initiative, providing support to increase healthy offerings among existing NC corner stores. It is unclear whether these changes will be associated with healthier purchases and healthier dietary patterns among customers. In our baseline evaluation of the NC HFSRP, we found a wide range of HFS and HEI scores, indicating that a wide range of healthy foods and beverages were supplied and purchased in the stores. When comparing store and customer characteristics, HFSRP and control stores and the respective customers in each group of stores were similar on many factors, but there were some differences (store-level HEI, racial composition, frequency of shopping at the store, soda and sweetened fruit drink consumption) which will be accounted for in follow-up evaluations. We also learned many valuable lessons which will be used to hone our follow-up data collection, including the need to deploy several teams of data collectors to different stores at the same time, and the need to be mindful of safety issues when selecting control stores.

To our knowledge, this is the first study to examine the associations between the healthfulness of the food supply in small stores and objectively-measured fruit and vegetable consumption using assessment of skin carotenoids with the validated RS Device. While we did not find statistically significant associations between HFS and HEI scores, or shopping patterns and RS Device-assessed skin carotenoids, our study demonstrates the feasibility of this objective, non-invasive measure of dietary intake in community-based natural experiments.

We found significant associations between shopping patterns and consumption, such that customers who had purchased fruits and vegetables at the store previously reported a higher fruit and vegetable intake compared to those who had not previously purchased fresh produce at the store. This indicates the potential benefit of the NC HFSRP, which has the goal of providing more healthy foods for customers to purchase. More research is needed to determine which elements of the corner store consumer food environment can facilitate and encourage healthier choices.

Customers who reported greater shopping frequency at the corner store where surveyed reported higher soda consumption compared to those reporting less frequent shopping. This corroborates prior studies showing that a majority of corner store purchases are unhealthy, processed foods and beverages [19,20]. This finding illustrates the need to assess the dose of food environment exposure, in order to understand how the frequency of contact with a particular food venue might influence purchasing and consumption.

This study is limited by the convenience sample, and the lack of ability to compare stores and customers who refused versus those that participated. As such, selection bias could be an issue, as those who are more interested in health and nutrition may have been more likely to participate. It is also possible that any relationships we found are a function of people with certain preferences selecting certain stores. This selection bias may have attenuated the relationship between healthfulness of purchases and dietary intake if a large portion of participants are already making healthy purchases. However, there was a range of HEI scores for purchases, and a range of dietary intake reported, suggesting that there were some with healthier purchase and consumption patterns than others. 

In some situations, we did not require the customer who completed the survey to have purchased food or beverage items, because graduate assistants had spent time traveling to the store and potentially eligible participants were purchasing lottery tickets only. Another potential limitation is that we also did not require participants to wash their hands before assessment with the RS Device, which could have contributed to inaccurate readings [44]. To minimize this potential limitation, we calibrated the device between each participant which increased accuracy of readings and used the mean of two RS Device readings in analyses. A further limitation is the use of self-reported height and weight to calculate BMI. Because individuals tend to underreport weight and overreport height, any associations related to BMI are likely to be biased downward. The analysis is also limited by defining “shopping patterns” by frequency of shopping at the store and whether the participant had purchased produce at the store, both of which are crude measures of shopping patterns. The time allotted for customer intercept surveys was limited, so these measures of shopping patterns were used to reduce survey participant burden. We did not include any measures of socioeconomic status in our analyses, which is a further limitation. This study is also limited by the cross-sectional design, which precludes any causality claims, as the exposure was assessed at the same time as the outcome. Strengths of the study include the use of objective data to assess purchases, the use of the ASA-24 for calculation of the HEI of store-level purchases, and the use of the RS Device (“Veggie Meter”) to assess customer-level fruit and vegetable consumption. The study is also strengthened by the large sample size of customers.

## 5. Conclusions

Our finding that customers who had previously purchased fruits and vegetables at corner stores had higher fruit and vegetable intake compared to those who had not previously purchased fresh produce illustrates that customers will purchase fruits and vegetables at corner stores. This notion is contrary to what some corner store owners and managers believe [46]. This finding should provide further support for healthy corner store initiatives like the NC HFSRP. Furthermore, the NC Legislature has allocated another $250,000 to the HFSRP for the state’s 2018 fiscal year, and future results of this evaluation effort are important to inform not only the NC Legislature, but other state-level policies to improve corner store food environments. In addition to state-level implications, our larger evaluation study has the potential to inform federal policy. Results from this study could be used to help to inform public comments for the U.S. Department of Agriculture (USDA)’s federal “Enhancing Retailer Standards in the Supplemental Nutrition Assistance Program” (SNAP Retailer Rule), and could help legislators understand whether policies to incentivize small stores to stock and promote healthier options are viable prevention strategies.

## Figures and Tables

**Table 1:** Healthy Food Supply Scores, Healthy Eating Index Scores among Healthy Food Small Retailer Program (HFSRP) and Control stores.

**HFSRP Store**	**First Healthy Food Supply Score**	**Second Healthy Food Supply Score**	**First Healthy Eating Index Score**	**Second Healthy Eating Index Score**
Store A	3.5	4.5	48.6	49.5
Store B	1.5	11.0	58.4	60.6
Store C	4.5	4.0	27.8	37.6
Store D	5.5	6.0	24.4	45.7
Store E	4.0	4.0	44.2	38.8
**Control Stores**	**First Healthy Food Supply Score**	**Second Healthy Food Supply Score**	**First Healthy Eating Index Score**	**Second Healthy Eating Index Score**
Store F	5.0	5.5	36.7	30.6
Store G	8.0	7.5	42.4	46.7
Store H *	Missing	Missing	32.6	Missing
Store I	5.0	3.0	34.8	51.0
Store J	3.0	Missing	28.7	41.0
Store K	1.0	0.5	36.6	34.6
Store L	5.0	5.0	41.3	28.9
Store M	1.5	2.0	35.5	31.6
Store N	7.5	7.5	32.2	43.4
Store O	1.5	Missing	39.9	18.8
Store P	4.0	Missing	25.9	32.9

* Data collectors felt unsafe conducting the audit and going back for the second visit to Store H.

**Table 2:** Demographic and dietary characteristics of customers from all stores (*n* = 479), and stratified by North Carolina Healthy Food Small Retailer Program stores and Control stores, and the *p*-value for the difference between HFSRP and Control store customers.

	Customers (*n* = 479) from all Stores		Customers (*n* = 161) from HFSRP Stores		Customers (*n* = 318) from Control Stores		*p*-value for Differences between HFSRP and Control Stores
Characteristic	Mean	Standard Deviation	Mean	Standard Deviation	Mean	Standard Deviation	
Age, years	43.3	15.1	43.0	15.1	43.4	15.1	0.79
	Number	Percent	Number	Percent	Number	Percent	
Sex (% female)	196	41.1	69	42.9	127	40.3	0.59
Race (% black/African American)	309	65.7	74	46.3	235	75.8	<0.001 **
Smoking status (% smokers)	228	55.2	71	45.8	157	60.9	0.003
Shopping frequency							<0.001 **
More than 1×/day	99	23.2	19	15.1	80	26.7	
1×/day	103	24.2	20	15.9	83	27.7	
5–6×/week	30	7.0	7	5.6	23	7.7	
3–4×/week	70	16.4	22	17.5	48	16.0	
1–2×/week	77	18.1	37	29.4	40	13.3	
1×/month	29	6.8	14	11.1	15	5.0	
Less than 1×/month	18	4.2	7	5.6	11	3.7	
Characteristic	Mean	Standard Deviation	Mean	Standard Deviation	Mean	Standard Deviation	
NCI Fruit, servings per day	2.2	2.4	2.2	2.6	2.2	2.2	0.89
NCI Vegetables, servings per day	2.7	2.1	2.6	2.1	2.7	2.1	0.91
NCI Fruits and Vegetables, servings per day	4.8	3.5	4.8	3.8	4.8	3.4	0.96
Skin carotenoids, RS Device, Intensities	234.2	86.2	236.6	76.5	232.9	91.0	0.65
Coefficient of variation for RS Device	0.067	0.081	0.055	0.057	0.074	0.091	0.007 **
Frequency of regular soda consumption, times per day	1.28	1.63	1.06	1.43	1.40	1.71	0.025 **
Frequency of sweetened fruit drink consumption, times per day	0.82	1.28	0.63	1.03	0.92	1.38	0.010 **
BMI	30.0	7.9	30.4	6.8	29.9	8.4	0.45
	Number	Percent	Number	Percent	Number	Percent	
Ever purchased fruits and vegetables from this store	104	21.9	36	22.5	68	21.7	0.834

** Indicates statistically significant differences between HFSRP and control store customers.

**Table 3:** Pearson’s correlation coefficients, 95% confidence intervals, and *p*-values between store-level Healthy Food Supply Scores, store-level Healthy Eating Index Scores, and individual-level fruit and vegetable, soda, and sweetened fruit drink consumption.

	Mean Vegetable Consumption, Self-Reported	Mean Fruit Consumption, Self-Reported	Mean Fruit and Vegetable Consumption, Self-Reported	Mean Objectively-Measured Fruit and Vegetable Consumption (RS Device)	Soda Consumption	Sweetened Fruit Drink Consumption
	0.015	−0.011	−0.001	0.087	−0.002	−0.041
HFS Score	(−0.081, 0.111)	(−0.108, 0.085)	(−0.097, 0.095)	(−0.009, 0.182)	(−0.096, 0.093)	(−0.135, 0.054)
	0.760	0.815	0.986	0.076	0.971	0.395
HEI Score	0.012	0.052	0.040	0.035	−0.065	−0.067
	(−0.08, 0.103)	(−0.039, 0.143)	(−0.051, 0.131)	(−0.057, 0.126)	(−0.153, 0.025)	(−0.155, 0.023)
	0.800	0.264	0.387	0.461	0.158	0.145

**Table 4:** Parameter estimates, standard errors, and *p*-values for associations between store-level Healthy Food Supply (HFS) Score, store-level Healthy Eating Index (HEI) score and individual-level self-reported and objectively-measured fruit and vegetable, soda, and sweetened fruit drink consumption., controlling for age, sex, and race.

Dependent Variable	Independent Variable	Parameter Estimate (95% Confidence Interval)	Standard Error	*p*-value
Fruit and vegetable consumption (Daily Servings)	HFS score	−0.013 (−0.160, 0.134)	0.075	0.860
Carotenoids (RS Device Intensities)	HFS score	2.207 (−2.798, 7.211)	2.545	0.387
Soda consumption (Daily Servings)	HFS score	−0.009 (−0.079, 0.062)	0.036	0.810
Sweetened fruit drink consumption (Daily Servings)	HFS score	−0.035 (−0.085, 0.014)	0.025	0.158
Fruit and vegetable consumption (Daily Servings)	HEI score	0.012 (−0.024, 0.048)	0.018	0.509
Carotenoids (RS Device Intensities)	HEI score	−0.315 (−1.619, 0.989)	0.664	0.635
Soda consumption (Daily Servings)	HEI score	−0.010 (−0.028, 0.008)	0.009	0.254
Sweetened fruit drink consumption (Daily Servings)	HEI score	−0.012 (−0.026, 0.001)	0.007	0.074
